# The joint impact of habitual exercise and glycemic control on the incidence of chronic kidney disease (CKD) in middle-aged and older males

**DOI:** 10.1186/s12199-017-0683-y

**Published:** 2017-11-06

**Authors:** Ryoma Michishita, Takuro Matsuda, Shotaro Kawakami, Satoshi Tanaka, Akira Kiyonaga, Hiroaki Tanaka, Natsumi Morito, Yasuki Higaki

**Affiliations:** 1 Department of Health Development, Institute of Industrial Ecological Sciences, University of Occupational and Environmental Health, Kitakyushu, Japan; 2 Fukuoka University Institute for Physical Activity, Fukuoka, Japan; 3 Department of Rehabilitation, Fukuoka University Hospital, Fukuoka, Japan; 4 Laboratory of Exercise Physiology, Faculty of Health and Sports Science, Fukuoka University, Fukuoka, Japan; 5 Fukuoka University Health Care Center, Fukuoka, Japan; 6 Department of Cardiology, Fukuoka University School of Medicine, Fukuoka, Japan

**Keywords:** Incidence of CKD, Habitual exercise, Glycemic control, Health checkup

## Abstract

**Background:**

This retrospective study evaluated the influence of the joint impact of habitual exercise and glycemic control on the incidence of chronic kidney disease (CKD) during a 6-year follow-up period in middle-aged and older males.

**Methods:**

The study population included 303 males without a history of cardiovascular disease, stroke, renal dysfunction, or dialysis treatment. Their lifestyle behaviors regarding exercise and physical activity were evaluated using a standardized self-administered questionnaire. The participants were divided into four categories according to the performance or non-performance of habitual exercise and the presence or absence of hyperglycemia.

**Results:**

After 6 years, 32 subjects (10.6%) developed CKD (estimated glomerular filtration rate < 60 ml/min/1.73 m^2^ and/or proteinuria). The cumulative incidence of CKD was significantly higher among subjects who did not perform habitual exercise and hyperglycemic subjects (log-rank test: *p* < 0.05, respectively). According to a Cox proportional hazards model, the hazard ratio (HR) for the incidence of CKD in subjects with a normal glucose tolerance (NGT) who did not perform habitual exercise (HR = 2.82, 95% confidence of interval (CI) = 1.07–7.36, *p* = 0.034) and that in hyperglycemic subjects who did not perform habitual exercise (HR = 5.89, 95% CI = 1.87–16.63, *p* = 0.003) were significantly higher in comparison to the subjects with a NGT who performed habitual exercise.

**Conclusions:**

These results suggest that the habitual exercise and good glycemic control and their combination were associated with the incidence of CKD.

## Background

The number of patients with end-stage renal disease (ESRD) in Japan is continuously increasing [1]. Chronic kidney disease (CKD) has been associated with the development of ESRD and cardiovascular disease (CVD) [2, 3]. At present, the large number of ESRD patients is thought to be related to an increase in the number of patients with CKD. The risk factors for CKD are reported to be caused by aging, hypertension, diabetes mellitus (DM), and metabolic syndrome [4–6]. In a recent study, we found that hypertension and hyperglycemia alone and in combination are independent risk factors for the incidence of CKD [7].

In addition to hypertension, type 2 DM, and metabolic syndrome, the incidence of CKD is also closely correlated with unhealthy lifestyle behaviors such as smoking, heavy alcohol intake, obesity, physical inactivity, and unhealthy diet [8–13]. In our previous cross-sectional and longitudinal studies [14–16], we found that the cumulative incidence of CKD significantly decreased as the number of healthy lifestyle behaviors increased. Lifestyle modifications, such as increased daily physical activity and adherence to habitual exercise, are the initial steps for the prevention of CVD [17]. In Japan, the number of patients with ESRD has been continuously increasing according to the increase in the incidence of type 2 DM [1]. The aim of glycemic control is not only to reduce the blood glucose level but also to prevent the development of CKD, ESRD, and CVD. Thus, we believe that the early stage of lifestyle counseling, with a particular focus on the regular performance of moderate exercise in order to improve hyperglycemia, is necessary for preventing the development of CKD, ESRD, and CVD. However, at present, the influence of habitual exercise and glycemic control alone and in combination on the incidence of CKD remains controversial.

We therefore hypothesized that a lack of habitual exercise and poor glycemic control and their combination might predict the incidence of CKD. Clarifying the influence of habitual exercise and glycemic control and their combination on the incidence of CKD may highlight the importance of CKD prevention. This retrospective study evaluated the influence of habitual exercise and glycemic control and their combination on the incidence of CKD during a 6-year follow-up period in middle-aged and older males.

## Methods

### Subjects

**Fig. 1:**
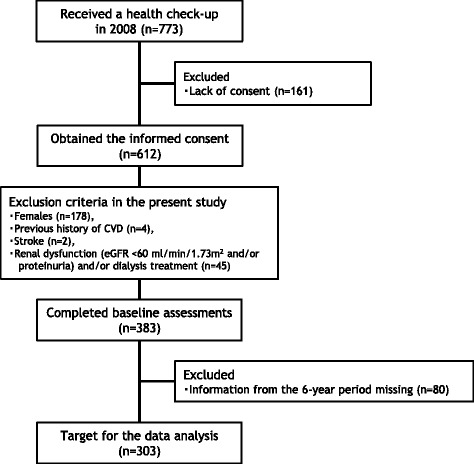
A flow chart of the subjects included in the study. CVD cardiovascular disease, eGFR estimated glomerular filtration rate

A total of 773 middle-aged and older adults received their periodic health checkup at a health-care center in Fukuoka University in 2008. Figure 1 shows a flow chart of the participants who were included in this study. Among the 612 subjects who provided their informed consent, 178 females were excluded to remove the influence of gender. Subjects with a previous history of CVD, such as angina and myocardial infarction (*n* = 4), stroke (*n* = 2), renal dysfunction (glomerular filtration rate estimated by the Japanese estimated glomerular filtration rate (eGFR) inference formula < 60 ml/min/1.73 m^2^, proteinuria, or both) [18], and/or dialysis treatment (*n* = 45), were also excluded from the analysis. A total of 303 males (age = 52.2 ± 6.7 years, body mass index (BMI) = 23.4 ± 2.8 kg/m^2^, serum creatinine = 0.84 ± 0.09 mg/dl, and eGFR = 77.0 ± 10.3 ml/min/1.73 m^2^) with no missing information over the previous 6 years were eligible for inclusion in the present study. In this study, subjects taking anti-hypertensive drugs, anti-hyperlipidemic agents, or hypoglycemic agents were included (anti-hypertensive drug users, *n* = 43; anti-hyperlipidemic agent users, *n* = 25; hypoglycemic agent users, *n* = 7).

All of the subjects gave their informed consent to participate after agreeing with the purpose, methods, and significance of the study. The study conformed to the Declaration of Helsinki guidelines and was approved by the Ethics Committee of Fukuoka University (No. 11-08-01).

### Blood sampling, blood pressure, and anthropometry measurement

Blood samples were collected early in the morning, after at least 12 h of fasting, from an antecubital vein by venipuncture. The blood samples were analyzed by Special Reference Laboratories (SRL, Inc., Tokyo, Japan). The serum creatinine, high-density lipoprotein cholesterol (HDL-C), and low-density lipoprotein cholesterol (LDL-C) levels were measured by the direct method. The triglyceride level was measured by the enzyme method. The plasma glucose level was measured by an ultraviolet/hexokinase method, and the hemoglobin A_1_c (HbA_1_c) level was measured by high-performance liquid chromatography. The HbA_1_c level was presented as the National Glycohemoglobin Standardization Program (NGSP) and International Federation of Clinical Chemistry and Laboratory Medicine (IFCC) values, which was calculated using the conversion equation for HbA_1_c derived from the Japan Diabetes Society (JDS): HbA_1_c (NGSP value; %) = 1.02 × JDS value (%) + 0.25% [19], and HbA_1_c (IFCC value; mmol/mol) = 10.93 × NGSP value (%) − 23.50 [20]. Hyperglycemia was defined based on the fasting plasma glucose and HbA_1_c levels according to the criteria of the Japan Diabetes Society as follows: fasting glucose ≥ 110 mg/dl and/or HbA_1_c (NGSP value) ≥ 6.5% (IFCC value > 48 mmol/mol) and/or the use of hypoglycemic drugs [21].

The grade of CKD was classified according to the eGFR level and the presence of proteinuria. The eGFR was calculated using the Japanese GFR inference formula, as follows: eGFR (ml/min/1.73 m^2^) = 194 × serum creatinine (mg/dl)^−1.094^ × age (years)^−0.287^ [22]. The GFR is a more accurate measure of the renal function than the serum creatinine level [23] and identifies patients who have mild renal impairment despite having normal or nearly normal creatinine levels. In addition, the eGFR is a strong predictor of cardiovascular events and is more useful for this purpose than the serum creatinine level [24, 25]. A urinalysis was performed using a dipstick, and the urine test results were classified as (−), (±), (1+), (2+), and (3+) [26]. In the present study, CKD was defined according to definition of the Japanese Society of Nephrology as follows: eGFR < 60 ml/min/1.73 m^2^, positive proteinuria (1+ or greater), or both [18]. The breakdown of the CKD grade of the subjects at baseline [18] was as follows: G1 (eGFR ≥ 90 ml/min/1.73 m^2^), *n* = 29 (9.6%), and G2 (eGFR 60–89 ml/min/1.73 m^2^), *n* = 274 (90.4%).

Blood pressure was measured in the right arm with the subject sitting in a chair, after at least 5 min of rest, and was expressed as an average of duplicate measurements. The height and body weight were measured, and the BMI was calculated as the ratio of the body weight (kg) to the height squared (m^2^). The waist circumference was measured at the level of the umbilicus.

### The assessment of lifestyle behaviors

The subjects’ lifestyle behaviors with regard to exercise, physical activity, and drinking and smoking habits were selected for based on the standardized self-administered questionnaire of the National Health Promotion Program, which was started in Japan in the fiscal year of 2008 and which aimed at preventing CVD, stroke, and metabolic syndrome [27, 28]. Our previous studies have shown that the combination of healthy lifestyle behaviors regarding exercise, physical activity, and diet is related to the incidence/prevalence of CKD in middle-aged and older males [14–16]. The subjects’ lifestyle behaviors regarding physical activity, exercise, eating style, and drinking and smoking habits were determined based on their responses to the following questionnaire items: habitual moderate exercise, ≥ 30 min at a time and ≥ 2 times per week (yes or no); physical activity equal to walking at least 1 h per day (yes or no); walking speed, compared with people of the same sex and age group (fast or slow); eating speed, compared with others (fast or slow); late night dinner, ≥ 3 times per week (yes or no); bedtime snacking, ≥ 3 times per week (yes or no); and skipping breakfast, ≥ 3 times per week (yes or no). The subjects’ drinking and smoking habits were assessed by the following questionnaire items (with “yes” or “no” responses): drinking habit (not drinking everyday) and smoking habit (recently not smoking). In this study, the subjects’ exercise habits were determined based on their responses to the following questionnaire items: habitual moderate exercise, ≥ 30 min at a time and ≥ 2 times per week (yes or no), physical activity equal to walking at least 1 h per day (yes or no), or both.

### Statistical analyses

In this study, subjects were followed for 6 years from October 2008 to October 2014. The biochemical analysis, blood pressure and anthropometry measurements, and assessment of lifestyle behaviors were conducted from baseline (2008) to end-point year (2014). The data were expressed as the mean and the standard deviation (SD). The StatView J-5.0 software package (SAS Institute, Cary, NC, USA) was used for all of the statistical analyses. In the present study, the subjects’ exercise and drinking and smoking habits were expressed as categorical variables, and the biochemical, blood pressure, and anthropometric indices were shown as continuous variables. As a result, the present study only analyzed data from participants who received their periodic health checkups during the 6-year study period. Inter-group comparisons were performed using the Mann-Whitney *U* test for continuous variables and the chi-square test for categorical variables. Within-group comparisons were determined using the one-way repeated-measures analysis of variance (ANOVA) and Scheffé’s method. The cumulative incidence of CKD was determined using the Kaplan-Meier survival curves and the log-rank test. A Cox proportional hazards model was used to predict the incidence of CKD using the parameters as categorical variables. This Cox proportional hazards model was adjusted for the following factors: age, BMI, eGFR, the use of anti-hypertensive drugs and anti-hyperlipidemic agents, and smoking and drinking habits at baseline. Probability values of < 0.05 were considered to indicate statistical significance.

## Results

After 6 years, the incidence of CKD (eGFR < 60 ml/min/1.73 m^2^ and/or proteinuria) was observed in 32 subjects (10.6%). After 6 years, the CKD grades of the subjects [18] were as follows: G1 (eGFR ≥ 90 ml/min/1.73 m^2^), *n* = 10 (3.3%); G2 (eGFR 60–89 ml/min/1.73 m^2^), *n* = 261 (86.1%); and G3a (eGFR 45–59 ml/min/1.73 m^2^), *n* = 32 (10.6%; including two with proteinuria). Table 1 shows the baseline characteristics of the subject who did and did not develop CKD. In the CKD group, the serum creatinine level, age, systolic blood pressure, diastolic blood pressure, fasting glucose, HbA_1_c levels, and rate of anti-hypertensive or anti-hyperlipidemic drug use were significantly higher and the eGFR, HDL-C levels, and the percentage of subjects who performed habitual exercise were significantly lower in comparison to the non-CKD group (*p* < 0.05, respectively). There were no significant differences in the other risk factors of the two groups.

**Table 1:** The baseline characteristics in subjects with and without the development of CKD

	All (*n* = 303)	Developed CKD (*n* = 32)	Did not develop CKD (*n* = 271)	*p* value
eGFR (ml/min/1.73 m^2^)	77.0 ± 10.3	66.8 ± 5.3	78.2 ± 10.1	< 0.0001
Classifications of CKD grade				
G1 (*n*, %; eGFR ≥ 90 ml/min/1.73 m^2^)	29 (9.6)	0 (0)	29 (10.7)	0.052
G2 (*n*, %; eGFR 60–89 ml/min/1.73 m^2^)	274 (90.4)	32 (100)	242 (89.3)	
Serum creatinine (mg/dl)	0.84 ± 0.09	0.93 ± 0.06	0.83 ± 0.09	< 0.0001
Age (years)	52.2 ± 6.7	54.6 ± 6.5	51.9 ± 6.7	0.030
Body weight (kg)	67.6 ± 9.3	67.7 ± 9.0	67.5 ± 9.4	0.902
BMI (kg/m^2^)	23.4 ± 2.8	23.3 ± 2.7	23.4 ± 2.8	0.921
Waist circumference (cm)	83.5 ± 7.6	84.6 ± 7.2	83.4 ± 7.6	0.379
SBP (mmHg)	126.8 ± 15.4	133.7 ± 15.2	126.0 ± 15.2	0.007
DBP (mmHg)	83.0 ± 10.4	86.6 ± 9.4	82.6 ± 10.5	0.038
LDL-C (mg/dl)	118.4 ± 25.2	119.4 ± 25.3	118.3 ± 25.1	0.819
HDL-C (mg/dl)	58.2 ± 13.3	53.7 ± 11.0	58.7 ± 13.5	0.043
Triglyceride (mg/dl)	115.0 ± 69.9	132.0 ± 124.0	113.0 ± 60.4	0.145
Fasting glucose (mg/dl)	100.5 ± 18.1	107.1 ± 30.1	99.7 ± 16.1	0.030
HbA_1_c (NGSP values; %)	5.6 ± 0.7	5.9 ± 0.9	5.6 ± 0.7	0.031
HbA_1_c (IFCC values; %)	38.0 ± 7.7	40.7 ± 9.6	37.7 ± 7.4	0.031
Smoking habit (yes/no; *n*, %)	63 (20.8)/240 (79.2)	5 (15.6)/27 (84.4)	58 (21.4)/213 (78.6)	0.446
Drinking habit (yes/no; *n*, %)	232 (76.6)/71 (23.4)	21 (65.6)/11 (34.4)	211 (77.9)/60 (22.1)	0.122
Anti-hypertensive drugs (yes/no; *n*, %)	43 (14.2)/260 (85.8)	9 (28.1)/23 (71.9)	34 (12.5)/237 (87.5)	0.017
Anti-hyperlipidemic agents (yes/no; *n*, %)	25 (8.3)/278 (91.7)	7 (21.9)/25 (78.1)	18 (6.6)/253 (93.4)	0.003
Hypoglycemic drugs (yes/no; *n*, %)	7 (2.3)/296 (97.7)	2 (6.3)/30 (93.7)	5 (1.8)/266 (98.2)	0.117
Exercise habit (yes/no; *n*, %)	148 (48.8)/155 (51.2)	10 (31.3)/22 (68.7)	138 (50.9)/133 (49.1)	0.035

The data are expressed as the mean ± standard deviation and the number of subjects. The CKD grades were defined according to the definitions of the Japanese Society of Nephrology [18]. The exercise habit was determined based on the subjects’ responses to the following questionnaire items: habitual moderate exercise ≥ 30 min at a time and ≥ 2 times per week and/or physical activity equal to walking at least 1 h per day

*CKD* chronic kidney disease, *eGFR* estimated glomerular filtration rate, *BMI* body mass index, *SBP* systolic blood pressure, *DBP* diastolic blood pressure, *LDL-C* low-density lipoprotein cholesterol, *HDL-C* high-density lipoprotein cholesterol, *HbA*_*1*_ *c* hemoglobin A_1_c, *NGSP* National Glycohemoglobin Standardization Program, *IFCC* International Federation of Clinical Chemistry and Laboratory Medicine

**Fig. 2:**
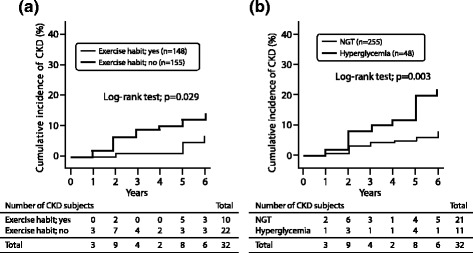
The cumulative incidence of CKD after a 6-year follow-up period in subjects according to the performance/non-performance of habitual exercise (**a**) and the presence/absence of hyperglycemia (**b**). NGT normal glucose tolerance

**Fig. 3:**
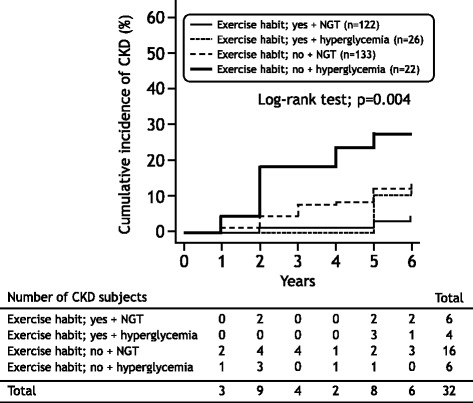
The cumulative incidence of CKD after 6 years of follow-up according to the combination of habitual exercise (performance/non-performance) and hyperglycemia (presence/absence). NGT normal glucose tolerance

Table 2 shows the baseline characteristics of the subject with and without habitual exercise and hyperglycemia. Among subjects who did not perform habitual exercise, systolic blood pressure (SBP), HbA_1_c level, and rate of anti-hyperlipidemic agent use were significantly higher and HDL-C level was lower in comparison to subjects who performed habitual exercise (*p* < 0.05, respectively). Among hyperglycemic subjects, age, SBP, diastolic blood pressure (DBP), triglyceride, fasting glucose, HbA_1_c levels, and rate of anti-hypertensive or hypoglycemic drug use were significantly higher and HDL-C level was lower in comparison to the normal glucose tolerance (NGT) subjects (*p* < 0.05, respectively). Figures 2 and 3 show the cumulative incidence of CKD over the 6-year follow-up period in subjects with and without habitual exercise and hyperglycemia. When the subjects were categorized according to the performance/non-performance of habitual exercise, the Kaplan-Meier survival curve showed that the cumulative incidence of CKD was significantly higher among subjects who did not perform habitual exercise (log-rank test: *p* = 0.029, Fig. 2a). When the subjects were categorized according to their glycemic control, the cumulative incidence of CKD among hyperglycemic subjects was significantly higher than that among NGT subjects (log-rank test: *p* = 0.003, Fig. 2b).

**Table 2:** The baseline characteristics in subjects with and without habitual exercise and hyperglycemia

	Habitual exercise		*p* value	Presence/absence of hyperglycemia		*p* value
	Yes (*n* = 148)	No (*n* = 155)		NGT (*n* = 255)	Hyperglycemia (*n* = 48)	
eGFR (ml/min/1.73 m^2^)	77.3 ± 10.5	76.7 ± 10.2	0.583	76.0 ± 9.5	77.2 ± 10.5	0.495
Classifications of CKD grade						
G1 (*n*, %; eGFR ≥ 90 ml/min/1.73 m^2^)	12 (8.1)	17 (11.0)	0.396	26 (10.2)	3 (6.3)	0.348
G2 (*n*, %; eGFR 60–89 ml/min/1.73 m^2^)	136 (91.9)	138 (89.0)		229 (89.8)	45 (93.7)	
Serum creatinine (mg/dl)	0.83 ± 0.10	0.85 ± 0.09	0.225	0.83 ± 0.09	0.84 ± 0.10	0.583
Age (years)	52.1 ± 6.5	52.3 ± 6.8	0.887	51.5 ± 6.7	55.6 ± 5.9	< 0.0001
Body weight (kg)	66.8 ± 9.9	68.4 ± 8.8	0.176	67.1 ± 9.1	69.5 ± 10.0	0.099
BMI (kg/m^2^)	23.1 ± 2.6	23.7 ± 2.8	0.094	23.3 ± 2.2	24.2 ± 2.6	0.137
Waist circumference (cm)	81.1 ± 8.0	85.4 ± 7.1	0.086	82.3 ± 7.5	85.6 ± 7.9	0.166
SBP (mmHg)	125.1 ± 15.6	129.6 ± 14.7	0.041	125.3 ± 14.6	134.2 ± 17.3	0.002
DBP (mmHg)	81.6 ± 10.4	83.8 ± 10.4	0.753	82.1 ± 10.2	85.9 ± 11.2	0.048
LDL-C (mg/dl)	117.0 ± 26.3	119.7 ± 24.1	0.346	118.9 ± 25.3	116.0 ± 24.5	0.456
HDL-C (mg/dl)	60.3 ± 13.2	56.1 ± 13.1	0.005	59.7 ± 13.1	54.3 ± 14.3	0.009
Triglyceride (mg/dl)	111.8 ± 69.1	117.9 ± 70.6	0.452	110.4 ± 67.9	138.2 ± 75.3	0.010
Fasting glucose (mg/dl)	97.8 ± 14.9	104.2 ± 20.8	0.146	95.0 ± 7.7	128.3 ± 27.9	< 0.0001
HbA_1_c (NGSP values; %)	5.5 ± 0.6	5.8 ± 0.8	0.036	5.4 ± 0.4	6.4 ± 1.3	< 0.0001
HbA_1_c (IFCC values; %)	35.5 ± 6.8	40.4 ± 8.4	0.031	36.3 ± 3.9	46.6 ± 13.9	< 0.0001
Smoking habit (yes/no; *n*, %)	30 (20.3)/118 (79.7)	33 (21.3)/122 (78.7)	0.827	56 (22.0)/199 (78.0)	7 (14.6)/41 (85.4)	0.195
Drinking habit (yes/no; *n*, %)	119 (80.4)/29 (19.6)	113 (72.9)/42 (27.1)	0.123	192 (75.3)/63 (24.7)	40 (83.3)/8 (16.7)	0.531
Anti-hypertensive drugs (yes/no; *n*, %)	19 (12.8)/129 (87.2)	24 (15.5)/131 (84.5)	0.509	29 (11.4)/226 (88.6)	14 (29.2)/34 (70.8)	0.002
Anti-hyperlipidemic agents (yes/no; *n*, %)	17 (11.5)/131 (88.5)	8 (5.2)/147 (94.8)	0.046	19 (7.5)/236 (92.5)	6 (12.5)/42 (87.5)	0.292
Hypoglycemic drugs (yes/no; *n*, %)	2 (1.4)/146 (98.6)	5 (3.2)/150 (96.8)	0.278	0 (0)/255 (100)	7 (14.6)/41 (85.4)	< 0.0001

The data are expressed as the mean ± standard deviation and the number of subjects

The abbreviations are the same as those in Table 1

The subjects were also divided into four categories based on the combination of the performance/non-performance of habitual exercise and the presence/absence of hyperglycemia. Table 3 shows the baseline characteristics of the subject according to the combination of habitual exercise (performance/non-performance) and hyperglycemia (presence/absence). Age, SBP, and rate of anti-hypertensive drug use were significantly higher, and HDL-C level was lower in the hyperglycemic subjects who did not perform habitual exercise than in the NGT subjects who performed habitual exercise (*p* < 0.05, respectively). Fasting glucose, HbA_1_c levels, and rate of hypoglycemic drug use were significantly higher in the hyperglycemic subjects who performed and did not perform habitual exercise than in the NGT subjects who performed and did not perform habitual exercise (*p* < 0.05, respectively). There were no significant differences in the other coronary risk factors among the four groups. The cumulative incidence of CKD in hyperglycemic subjects who did not perform habitual exercise was significantly higher than that among NGT subjects who did not perform habitual exercise, hyperglycemic subjects who performed habitual exercise, and NGT subjects who performed habitual exercise (log-rank test: *p* = 0.004, Fig. 3). In a univariate analysis, the non-performance of habitual exercise was significantly associated with the incidence of CKD in the subjects with a NGT (HR = 2.77, 95% CI = 1.08–7.08, *p* = 0.033) and in the hyperglycemic subjects (HR = 6.77, 95% CI = 2.18–18.02, *p* = 0.001). Furthermore, after adjusting for age, BMI, eGFR, the use of anti-hypertensive and anti-hyperlipidemic drugs, and smoking and drinking habits at baseline, the non-performance of habitual exercise remained associated with the incidence of CKD in the subjects with NGT (HR = 2.82, 95% CI = 1.07–7.36, *p* = 0.034) and the hyperglycemic subjects (HR = 5.89, 95% CI = 1.87–16.63, *p* = 0.003, Table 4).

**Table 3:** The baseline characteristics in subjects according to the combination of habitual exercise (performance/non-performance) and hyperglycemia (presence/absence)

	Performance of habitual exercise		Non-performance of habitual exercise		*p* for ANOVA
	NGT (*n* = 122)	Hyperglycemia (*n* = 26)	NGT (*n* = 133)	Hyperglycemia (*n* = 22)	
eGFR (ml/min/1.73 m^2^)	77.2 ± 10.7	78.6 ± 10.8	76.9 ± 10.4	73.9 ± 7.2	0.443
Classifications of CKD grade					
G1 (*n*, %; eGFR ≥ 90 ml/min/1.73 m^2^)	10 (8.2)	3 (11.5)	16 (12.0)	0 (0)	0.247
G2 (*n*, %; eGFR 60–89 ml/min/1.73 m^2^)	112 (91.8)	23 (88.5)	117 (88.0)	22 (100)	
Serum creatinine (mg/dl)	0.84 ± 0.10	0.81 ± 0.10	0.85 ± 0.09	0.85 ± 0.08	0.363
Age (years)	50.5 ± 6.8	54.8 ± 6.4	52.6 ± 6.5	56.0 ± 5.3 ^a^	0.002
Body weight (kg)	67.4 ± 9.8	69.3 ± 10.4	67.0 ± 8.5	69.4 ± 10.1	0.515
BMI (kg/m^2^)	23.0 ± 2.4	23.7 ± 2.2	23.3 ± 2.9	23.4 ± 2.4	0.937
Waist circumference (cm)	83.2 ± 8.2	84.3 ± 7.1	83.3 ± 6.8	85.5 ± 9.1	0.566
SBP (mmHg)	123.6 ± 15.1	132.7 ± 13.0	127.4 ± 14.1	134.5 ± 20.5 ^a^	0.001
DBP (mmHg)	82.3 ± 10.2	85.6 ± 12.4	82.8 ± 10.2	85.4 ± 10.5	0.341
LDL-C (mg/dl)	117.3 ± 26.2	117.7 ± 25.3	120.7 ± 24.3	114.6 ± 24.0	0.486
HDL-C (mg/dl)	60.7 ± 12.8	56.8 ± 13.0	55.8 ± 14.8	54.3 ± 14.3 ^a^	0.032
Triglyceride (mg/dl)	109.2 ± 67.7	111.1 ± 68.0	139.4 ± 76.3	141.8 ± 77.4	0.052
Fasting glucose (mg/dl)	94.1 ± 9.8	126.5 ± 26.4 ^a, b^	96.6 ± 7.4	129.3 ± 28.6 ^a, b^	< 0.0001
HbA_1_c (NGSP values; %)	5.5 ± 0.4	6.3 ± 1.3 ^a, b^	5.5 ± 0.4	6.4 ± 0.9 ^a, b^	< 0.0001
HbA_1_c (IFCC values; %)	36.2 ± 3.8	46.3 ± 14.8 ^a, b^	36.6 ± 4.4	46.8 ± 10.2 ^a, b^	< 0.0001
Smoking habit (yes/no; *n*, %)	27 (22.1)/95 (77.9)	3 (11.5)/23 (88.5)	29 (21.8)/104 (78.2)	4 (18.2)/18 (81.8)	0.630
Drinking habit (yes/no; *n*, %)	100 (82.0)/22 (18.0)	19 (73.1)/7 (26.9)	96 (72.2)/37 (27.8)	17 (77.3)/5 (22.7)	0.102
Anti-hypertensive drugs (yes/no; *n*, %)	16 (13.1)/106 (86.9)	3 (11.5)/23 (88.5)	15 (11.3)/118 (88.7)	9 (40.9)/13 (59.1)	0.003
Anti-hyperlipidemic agents (yes/no; *n*, %)	14 (11.5)/108 (88.5)	3 (11.5)/23 (88.5)	5 (3.8)/128 (96.2)	3 (13.6)/19 (86.4)	0.127
Hypoglycemic drugs (yes/no; *n*, %)	0 (0)/122 (100)	2 (7.7)/24 (92.3)	0 (0)/133 (100)	5 (22.7)/17 (77.3)	< 0.0001

The data are expressed as the mean ± standard deviation and the number of subjects

The abbreviations are the same as those in Table 1

^a^ *p* < 0.05, compared to the NGT subjects who performed habitual exercise

^b^ *p* < 0.05, compared to the NGT subjects who did not perform habitual exercise

**Table 4:** The influence of the combination of an exercise habit and the glycemic control on the incidence of CKD

	Total	Developed CKD (*n*, per 10,000 person-years)	Developed proteinuria (*n*, per 10,000 person-years)	Univariate model		Multivariable model	
				Hazard ratio (95% CI)	*p* value	Hazard ratio (95% CI)	*p* value
Combined with exercise habit and glycemic control states							
Exercise habit; yes + NGT	122	6 (82.0)	0	1.00 (Ref.)	–	1.00 (Ref.)	–
Exercise habit; yes + hyperglycemia	26	4 (256.4)	0	3.29 (0.93–9.66)	0.065	2.50 (0.69–9.14)	0.164
Exercise habit; no + NGT	133	16 (200.5)	1 (12.5)	2.77 (1.08–7.08)	0.033	2.82 (1.07–7.36)	0.034
Exercise habit; no + hyperglycemia	22	6 (454.5)	1 (75.8)	6.77 (2.18–18.02)	0.001	5.89 (1.87–16.63)	0.003

The data are expressed as the hazard ratio (95% confidence interval (CI)). In this analysis, the lack of an exercise habit and the prevalence of hyperglycemia at baseline were dependent variables and the incidence of CKD was an independent variable. The multivariable model was adjusted for age, BMI, eGFR, the use of anti-hypertensive drugs and anti-hyperlipidemic agents, and smoking and drinking habits at baseline

## Discussion

The major findings of our study were that the non-performance of habitual exercise and hyperglycemia were significantly associated with the incidence of CKD. In addition, the relative risk for the incidence of CKD among hyperglycemic subjects who did not perform habitual exercise was significantly higher than that among NGT subjects who performed habitual exercise. The results of the present study suggest that a lack of habitual exercise and poor glycemic control alone and in combination are independent risk factors for the incidence of CKD.

Adherence to habitual exercise is well known to be related to a decreased incidence of cardiovascular morbidity and mortality [29–32]. Additionally, it is also well known that poor glycemic control is an important independent risk factor for the development of CVD [33]. We hypothesize that similar mechanisms underlie the relationship between the non-performance of habitual exercise and poor glycemic control and their combination with the incidence of CKD and that adherence to habitual exercise and good glycemic control and their combination plays a pivotal role in the prevention of cardiovascular events. Dunkler et al. [34] investigated the effects of modifiable lifestyle factors on the incidence of CKD and mortality in individuals with type 2 DM and showed that there was a significant increase in the incidence of CKD development and in the mortality rate as the lifestyle scores decreased. Ricardo et al. [35] evaluated the influence of four health lifestyle factors (regular physical activity, maintaining a reasonable body weight, not smoking, and enjoying a healthy eating style) on the incidence of CKD progression, cardiovascular events, and all-cause mortality and found that the incidence of CKD and atherosclerotic events and the rate of all-cause mortality were significantly reduced as the healthy lifestyle score increased. A previous meta-analysis [11] reported that a lack of habitual exercise and decreased physical activity influenced the incidence of CKD. Those authors showed that the incidence of CKD, cardiovascular events, and all-cause mortality were significantly reduced as the healthy lifestyle behaviors including exercise and physical activity increased. Our recent cross-sectional and longitudinal studies [14–16] also found that an increase in healthy lifestyle behaviors, especially those related to a lack of habitual exercise, may be predictive factors for the incidence of CKD. Furthermore, we also demonstrated that hypertension and hyperglycemia and their combination may be independent risk factors for the incidence of CKD [7]. At present, the number of patients with ESRD in Japan is continuously increasing according to the increase in the incidence of type 2 DM [1]. Thus, our current findings support the possibility that adherence to habitual exercise and good glycemic control and their combination are important for preventing the development of CKD, ESRD, and CVD, and we consider that the early stage of lifestyle counseling, with a particular focus on the importance of regularly performing moderate exercise to improve hyperglycemia, is necessary in order to prevent the development of CKD, ESRD, and CVD.

In the present study, the NGT subjects who did not perform habitual exercise, but not hyperglycemic subjects who performed habitual exercise, were significantly associated with the incidence of CKD. However, at present, the influence of habitual exercise and glycemic control states alone and in combination on the incidence of CKD has not yet been investigated. Nagasawa et al. [36] investigated the association between exercise and the prevalence of proteinuria and kidney dysfunction and the attenuation by obesity and demonstrated habitual exercise may ameliorate the prevalence of proteinuria and kidney dysfunction and a high BMI may attenuate this amelioration in male subjects. On the other hand, Manson et al. [37] examined that the relationships between physical activity level and cardiovascular events and found that daily walking, especially vigorous exercise, was associated with a lower risk of cardiovascular events independent of age and BMI. Lee et al. [38] reported the combined associations of changes in cardiorespiratory fitness and the BMI with all-cause and cardiovascular mortality. Those authors showed that maintaining or improving fitness is associated with a lower risk of all-cause and cardiovascular mortality. Thus, at present, the non-performance of habitual exercise, decreased physical activity, and cardiorespiratory fitness itself are thought to be related to the development of cardiovascular morbidity and mortality independent of aging and obesity. According to these findings, a lack of habitual exercise may be a risk factor for detecting the development of CKD, ESRD, and CVD, independent of glycemic control, at least in the population of the present study because the NGT subjects who did not perform habitual exercise, but not hyperglycemic subjects who performed habitual exercise, were significantly correlated with the incidence of CKD.

### Study limitations and clinical implications

There are several limitations associated with this study. First, the limited study population resulted in a small number of male subjects, who were predominantly middle-aged and older, and those who did not have any health complications. Thus, there was a potential selection bias in this study, as our limited study population may have included more CKD subjects with a slowly declining renal function than CKD subjects with rapid deterioration. As such, it remains unclear whether or not our findings are generalizable to females, patients with ESRD, or individuals with other complications. Second, although this study was performed within the constraints of the health checkup, it was not possible to clarify the causality of the incidence of CKD with a lack of habitual exercise and poor glycemic control. Third, the indices of glucose tolerance in the present study were evaluated based on the fasting glucose and HbA_1_c levels. An impaired glucose tolerance, as measured with an oral glucose tolerance test, was associated with an increased risk of CVD in comparison to an impaired fasting glucose level [33], which could not be evaluated in our subjects. Finally, we calculated the eGFR using the Japanese GFR inference formula [22] and used proteinuria as an index of the renal function. Unfortunately, in the present study, the causality of proteinuria with the lack of habitual exercise and poor glycemic control could not be clarified because of the small number of subjects with proteinuria. To fully clarify the influence of the non-performance of habitual exercise and hyperglycemia and the combination thereof on the renal function, other indices of the renal function, such as urinary protein excretion, microalbuminuria, or cystatin C, should be simultaneously assessed. However, we were unable to measure any additional markers of the renal function because this study was performed within the constraints of the health checkup.

However, despite these limitations, the present study is the first to elucidate the joint impact of habitual exercise and glycemic control on the incidence of CKD over a long follow-up period. We believe that our results support the notion that habitual exercise and good glycemic control and their combination can prevent the incidence of CKD. In our recent studies, we observed that the combination of healthy lifestyle behaviors with regard to exercise, physical activity, and diet was related to the incidence/prevalence of CKD in middle-aged and older males [14–16]. Furthermore, we found that hypertension and hyperglycemia and their combination may be associated with the incidence of CKD [7]. Thus, the results of the present study show that the non-performance of habitual exercise, hyperglycemia, and the combination of these factors are related to the incidence of CKD. This may support the hypothesis that the non-performance of exercise and poor glycemic control lead to an increase in the incidence of CVD and the development of ESRD. Given our results, we believe that providing lifestyle counseling at an early stage, with a particular focus on the role of regular moderate exercise in improving hyperglycemia, is necessary to prevent the incidence of CKD, ESRD, and CVD. Further investigations in larger study populations, including subjects with other complications, are needed to more precisely clarify the mechanisms, clinical implications, and associations of habitual exercise, glycemic control, and their combination with the incidence of CKD following long-term intervention.

## Conclusions

This retrospective study examined the influence of the joint impact of habitual exercise and glycemic control on the incidence of CKD in middle-aged and older males over a 6-year follow-up period. We demonstrated that the cumulative incidence of CKD among the NGT subjects who did not perform habitual exercise and hyperglycemic subjects who did not perform habitual exercise was significantly higher than in NGT subjects who performed habitual exercise. These results suggest that adherence to habitual exercise and the maintenance of good glycemic control and their combination were associated with the incidence of CKD.

## Abbreviations

ANOVA: Analysis of variance
BMI: Body mass index
CI: Confidence of interval
CKD: Chronic kidney disease
CVD: Cardiovascular disease
DBP: Diastolic blood pressure
DM: Diabetes mellitus
eGFR: Estimated glomerular filtration rate
ESRD: End-stage renal disease
HbA_1_c: Hemoglobin A_1_c
HDL-C: High-density lipoprotein cholesterol
HR: Hazard ratio
IFCC: International Federation of Clinical Chemistry and Laboratory Medicine
LDL-C: Low-density lipoprotein cholesterol
NGSP: National glycohemoglobin standardization program
NGT: Normal glucose tolerance
SBP: Systolic blood pressure
